# Complete Response and Fatigue Improvement With the Combined Use of Cyclophosphamide and Quercetin in a Patient With Metastatic Bladder Cancer: A Case Report

**DOI:** 10.1097/MD.0000000000002598

**Published:** 2016-02-08

**Authors:** Giuseppe Di Lorenzo, Martina Pagliuca, Teresa Perillo, Aquilino Zarrella, Antonio Verde, Sabino De Placido, Carlo Buonerba

**Affiliations:** From the Medical Oncology Unit, Department of Clinical Medicine, Federico II University, Naples, Italy.

## Abstract

Bladder cancer is a major cause of cancer-related mortality, with an estimated 74,000 new cases and 16,000 deaths in the United States in 2015. In patients with metastatic disease, vinflunine and taxanes are the most widely used chemotherapy agents in the second-line setting after failure of platinum-based treatment. Cyclophosphamide has been used in combination with paclitaxel in urothelial carcinoma of the bladder, but there are no data about the effectiveness of cyclophosphamide administered as a single agent.

We here describe the first case of an advanced bladder cancer patient suffering from grade 2 fatigue.

He benefited from administration of third-line single-agent metronomic oral cyclophosphamide plus oral doses of quercetin. A complete, prolonged radiologic response according to the RECIST criteria 1.1 was achieved with minimal toxicity and an improvement in fatigue.

Further studies are required to assess the potential benefits associated with the combined use of cyclophosphamide plus quercetin in advanced bladder cancer patients.

## INTRODUCTION

Bladder cancer is a major cause of cancer-related mortality, with an estimated 74,000 new cases and 16,000 deaths in the United States in 2015.^1^ Risk factors include smoking, occupational exposure to polycyclic aromatic hydrocarbons and aromatic amines,^2^ and, possibly, environmental pollution,^3^ whereas fruits and vegetables intake may exert a protective effect.^4^ Stage is a major prognostic factor, with a 5-year survival of 70% and 4%, respectively, in patients with localized bladder cancer and in patients with metastatic disease.^1^ Presently, surgery remains the mainstay of treatment of localized disease, although the risk of recurrence in cystectomy patients is reported to be as high as 70%.^5^ In patients with metastatic disease, chemotherapy is able to provide a modest survival prolongation, with platinum-based first-line treatment being associated with a median survival of only 14 to 15 months.^6^ A number of different chemotherapy regimens have been tested in the salvage setting after failure of a platinum-based regimen, but only vinflunine yielded a benefit versus best supportive care alone in a randomized-controlled phase III trial, with a significant 2.5 month survival improvement in the eligible population (6.8 vs 4.3 months).^7^ Taxanes are also widely used for the second-line treatment of bladder cancer on the grounds of the results achieved in a number of phase II studies.^8,9^ Paclitaxel and docetaxel can be administered either alone or in combination with agents such as gemcitabine, cyclophosphamide, ifosfamide, and epirubicin, and an improvement in survival has been reported with taxane-based combination chemotherapy versus single-agent taxane.^10^ Only a few studies with small samples sizes (<25 patients) have been conducted to explore the efficacy of chemotherapy in the third-line setting,^11–13^ with modest results in terms of progression-free survival (2–5 months) and overall survival (6–8 months). Cyclophosphamide, a nitrogen mustard alkylating agent, shows antiangiogenic and immune-stimulating activity with minimal toxicity when used as part of a metronomic regimen.^14^ Although metronomic cyclophosphamide has been used in combination with paclitaxel in urothelial carcinoma of the bladder, there are no data about its effectiveness as a single agent. We here describe the case of a patient with metastatic urothelial carcinoma of the bladder, who received third-line single-agent metronomic oral cyclophosphamide and consumed daily doses of quercetin reporting an antifatigue effect, a complete radiologic response according to the RECIST criteria 1.1,^15^ a prolonged progression-free survival and minimal toxicity.

## CASE REPORT

**FIGURE 1:**
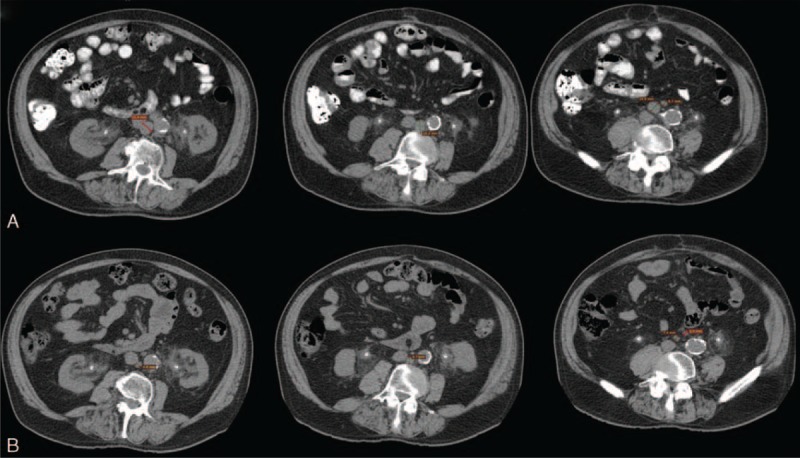
CT scan showing target lymphnodes at baseline (A) and after 6 months of treatment (B). CT = computed tomography.

The patient, a male former smoker (40 package-years) born in 1932, presented a past medical history of peptic ulcer, hypertension, and thoracic aortic aneurysm. After the first diagnosis of transitional cell bladder cancer (pT1 G1) in January 2007, the patient was treated with TURB plus intravescical mytomicin C. The subsequent follow-up was inconsistent due to poor patient compliance. In November 2012, the patient reported macrohematuria, followed by acute urinary retention and bladder catheterization the following month. A whole-body CT scan with and without contrast showed the presence of an intravescical tumor mass and widespread bilateral lymph node metastases in the obturator fossae and along the iliac vases (short axis, 2.3 cm max). In February 2013, the patient underwent cystectomy plus iliac and obturator lymphadenectomy. Histology examination of the surgical specimen showed a high-grade urothelial carcinoma infiltrating the perivescical fat, with extensive lymphnode involvement (pT3 N2). In April 2013, whole-body CT scan with and without contrast showed bilateral persistent pelvic and retroperitoneal lymph node metastases in the obturator fossa (1.5 cm), along the right iliac vases (right, 3.3 cm max) and in the interaortocaval space (1.5 cm max). First-line treatment for metastatic disease consisted of 5 cycles of cisplatin plus gemcitabine, which were administered since April, 2013, until August, 2013. After the 5th cycle, the patient suffered from kidney damage, so he received carboplatin plus gemcitabine in September, 2013. In October 2013, CT scan showed persistence of enlarged lymphnodes in the obturator fossa (2 cm max), so the patient received paclitaxel-based salvage chemotherapy. Paclitaxel was preferred over vinflunine, because the patient had been suffering from severe constipation for the past 2 weeks at the time of initiation of second-line paclitaxel. After 4 administrations of weekly paclitaxel, he refused any further intravenous chemotherapy. In February, 2014, CT scan showed pathologic enlargement of the lymphnodes in the obturator fossa (bilateral, 2 cm), as well as in the interaortocaval (2 cm max) and in the iliac (right, 2 cm max) space, measured according to the RECIST 1.1 criteria. Bone marrow, heart, lung, and hepatic function was within normal limits, consistently with the patient's age and medical history, but the glomerular filtration rate was moderately reduced (42 mL/min; Cockcroft-Gault). The patient, who presented grade 2 fatigue (fatigue not relieved by rest and limiting instrumental activities of daily living) and an ECOG PS of 2, was started on oral cyclophosphamide (100 mg a day) after obtaining written informed consent to treatment. In an attempt to relieve fatigue, the patient was also suggested to consume quercetin (1 g a day) as a nutritional dietary supplement. In April 2014, the patient showed a partial radiological response, whereas fatigue and PS had improved to grade 1 and ECOG 1, respectively. In July 2014, a complete response was reported on CT scan according to the RECIST criteria 1.1, with shrinkage of all previously enlarged lymph nodes (short axis, max 1 cm) (Figure 1). The patient had increased consumption of quercetin (2 g/a day), although he was discouraged to consume daily doses >1 g. During the 56 weeks administration period, the patient continued to receive cyclophosphamide and kept on consuming ∼2 g a day of quercetin, with no significant adverse events. CT scans were repeated at 3 month intervals. Radiological progression was reported in March 2015, with widespread pelvic and abdominal lymphnode disease. Although the patient showed grade 2 anemia and grade 3 fatigue and his PS was 2, he consented to receive intravenous weekly paclitaxel, but had to interrupt treatment after 1 cycle for persisting grade 3 fatigue and grade 3 anemia. He died in November, 2015.

## DISCUSSION

In the case presented here, the use of single-agent cyclophosphamide was associated with a confirmed radiological response and prolonged progression-free survival with minimal toxicity and excellent compliance in an elderly patient who had been pretreated with 2 lines of chemotherapy for metastatic disease. The patient had received platinum-based chemotherapy and taxane agent paclitaxel. Paclitaxel, which is accepted as a standard second-line treatment option,^16^ was preferred to vinflunine as vinflunine was associated with grade 3–4 constipation in 16.1% of patients enrolled in the phase III trial on vinflunine versus best supportive care.^7^ We are unaware of published data regarding the use of single-agent cyclophosphamide in urothelial carcinoma. In a phase I/II trial exploring the use of paclitaxel plus cyclophosphamide, Di Lorenzo^9^ et al that concluded that 50 mg was the maximum tolerated daily dose of oral cyclophosphamide administered on days 1 to 7 concomitantly with 175 mg/m^2^ of intravenous paclitaxel on day 1. In the 32 patients enrolled in the phase II part of the trial, the median time to progression was 5 months (95% CI, 2 –7.5 months), whereas the median OS was 8 months (95% CI, 4–14 months), with grade 1/2 vomiting, peripheral neuropathy, and neutropenia reported in 34%, 25%, and 31% of cases, respectively, and grade 3–4 neutropenia occurring in 34.5 % of patients. Of note, no complete response was reported, with approximately one-third of patients showing a partial radiological response. In a separate phase II trial,^17^ 46 patients diagnosed with bladder or upper urinary tract cancer were treated with paclitaxel+cyclophosphamide at the same doses used by Di Lorenzo et al, although cyclophosphamide was administered for 3 weeks after the first cycle. The median time to progression is 3 months (95% CI 1.7–4.3 months), with an objective response rate of 33.3% and a median OS of 6.3 months (95% CI 4.6–8.0 months). Of note, 1 patient reported a complete radiologic response. Different from the results by Di Lorenzo et al, grade ≥ 3 neutropenia occurred only in 2 patients (6%), with one of them developing febrile neutropenia. The most clinically significant nonhematologic toxicities reported were grade 1–2 myalgia (28%), peripheral sensory neuropathy (56%), and fatigue (35%). The toxicity profile associated with the use of single-agent cyclophosphamide in the case presented here compares favorably with that of paclitaxel+cyclophosphamide discussed above. The patient did not report any adverse event, and he also experienced improvement of fatigue, which we speculate it may be associated with quercetin consumption due to the quercetin effect on glucose metabolism and inflammation. Cancer-related fatigue has been associated with a number of causative factors, including inflammatory processes, disruption of the hypothalamus-pituitary-adrenal gland axis, as well as metabolic disturbances with decreased ATP production.^18^ Similar to other flavonols, quercetin is able to decrease TNF-alfa and IL-6 serum levels.^19^ Preclinical data show that quercetin is able to boost cellular metabolism by increasing the activity of AMPK, a key energy-sensor enzyme that stimulates skeletal muscle fatty acid oxidation and muscle glucose uptake. In 1 in vitro study conducted in a mouse myoblast cell line exposed to 50 or 100 μM quercetin, stimulation of AMPK resulted in decreased activity of acetyl-CoA carboxylase and increased fatty acid transportation into the mitochondria.^20^ In mice randomly assigned to either placebo or 12.5 mg/kg quercetin or 25 mg/kg quercetin and undergoing a treadmill performance run to fatigue, quercetin increased markers of mitochondrial biogenesis (PGC-1alpha, cytochrome *c*) and improved both maximal endurance capacity and voluntary wheel-running activity.^21^ These results paralleled those obtained in clinical trials, with 1 meta-analysis showing that quercetin supplementation was associated with increased endurance performance.^22^

In the case presented here, we obtained a marked improvement in fatigue and a complete and prolonged radiologic response in a patient treated with third-line chemotherapy. Only a few studies have been conducted in this setting. Matsumoto^11^ et al evaluated the efficacy of gemcitabine plus nedaplatin in 10 patients with metastatic urothelial carcinoma who had been previously treated with methotrexate, vinblastine, doxorubicin, and cisplatin, followed by gemcitabine and paclitaxel. The median overall survival was 8.8 months, whereas the median progression-free survival was 5 months. Similarly, the use of pegylated liposomal doxorubicin as a third-line chemotherapy was associated with a median progression-free survival and overall survival of 4.1 and 6.3 months in 23 patients with advanced urothelial carcinoma, whereas median time to progression and overall survival were 2 and 7.3 months, respectively, in 13 patients treated with third-line gemcitabine.^12,13^ These findings confirm the poor prognosis of patients treated for advanced urothelial carcinoma in the third-line setting and highlight the need for novel therapeutic options. Immunotherapy holds a great promise for advanced urothelial carcinoma.^23^ As an example, a novel anti-PD-1 agent was associated with clinical activity in a phase I study in metastatic bladder cancer.^24^ Low-dose metronomic cyclophosphamide, which can induce antitumor in vivo immunity,^25^ may synergize with novel immunotherapy agents^23^ via depletion of regulatory T cells.^26^

## CONCLUSIONS

This case shows that a novel combination of metronomic cyclophosphamide plus quercetin may have an antineoplastic and antifatigue effect with an excellent safety profile. Complete responses are rare in bladder cancer patients, whereas very few agents have a demonstrated antifatigue effect. Further studies are required to assess the potential benefits associated with the combined use of cyclophosphamide plus quercetin in advanced bladder cancer patients.
